# Myophosphorylase Knock Out Prevents the Exaggerated Exercise Pressor Reflex in Rats With Simulated Peripheral Artery Disease

**DOI:** 10.1111/apha.70172

**Published:** 2026-02-16

**Authors:** Guillaume P. Ducrocq, Laura Anselmi, Victor Ruiz‐Velasco, Marc P. Kaufman

**Affiliations:** ^1^ Heart and Vascular Institute Penn State College of Medicine Hershey Pennsylvania USA; ^2^ Faculté de Médecine, UR3072 «Mitochondrie, Stress Oxydant et Plasticité Musculaire» Université de Strasbourg Strasbourg France; ^3^ Department of Anesthesiology and Perioperative Medicine Penn State College of Medicine Hershey Pennsylvania USA

**Keywords:** autonomic function, blood pressure, chronic ischemia, lactate, metaboreflex, muscle acidosis, peripheral artery disease, sympathetic activity

## Abstract

**Aim:**

Controversy exists on which metabolites determine the exaggerated exercise pressor reflex (EPR) in peripheral artery disease (PAD). In decerebrated rats, we investigated the role played by lactate and hydrogen ions in a model of PAD, which was simulated by ligating the femoral artery for 72 h before the start of the experiment.

**Methods:**

Production of lactate and hydrogen ions by the contracting hindlimb muscles was manipulated by knocking out the myophosphorylase gene (pygm). In both knockout (pygm^−/−^; *n* = 13; 6‐females) and wild‐type rats (pygm^+/+^; *n* = 14; 7‐females), the EPR was evoked by statically contracting the triceps‐surae muscles. Blood pressure, tension, and renal sympathetic nerve activity were measured. Responsiveness of the metabolic component of the EPR was evaluated by intra‐arterial injections of lactic acid and diprotonated phosphate solutions. Responsiveness of the mechanical component of the EPR was evaluated by stretching the calcaneal tendon. In each rat, the pressor responses evoked from the freely perfused triceps‐surae muscles were compared to those evoked from the contralateral ischemic triceps‐surae muscles.

**Results:**

In pygm^+/+^ rats whose femoral artery was ligated, static contraction, lactic‐acid injection and diprotonated phosphate injection evoked pressor responses that were 88%, 22%, and 58% greater than those evoked from muscles whose femoral arteries were freely perfused. In pygm^−/−^ rats, ligation of the femoral artery for 72 h had no effect. In both groups, 72 h of femoral artery ligation exacerbated the pressor response to passive stretch.

**Conclusion:**

Lactate and hydrogen‐ions accumulation in contracting myocytes plays a key role in exaggerating the metabolic component of the EPR evoked from hindlimb muscles with chronically‐ligated femoral arteries.

## 1 Introduction

Peripheral artery disease is a vascular pathology that affects more than 200 million patients worldwide [1]. It is characterized by the progressive narrowing of arterial lumen from plaque buildup on the vessel walls [2]. As a result, blood and oxygen delivery to contracting muscles is impaired [2, 3], intramuscular metabolites and inflammatory by‐products are accumulated at a faster rate and in greater concentrations than during normal exercise [4, 5, 6]. Even at mild exercise intensity, peripheral artery disease exaggerates sympathetic and arterial blood pressure responses to exercise [7, 8, 9], thereby markedly increasing the risk of life‐threatening hypertensive events and severely impairing patients' quality of life [2]. Elucidating the mechanisms underlying this exaggerated pressor response to exercise is therefore crucial for developing better treatments and ultimately improving both survival and quality of life in these patients.

Among the mechanisms that are responsible for regulating the hemodynamic response to exercise, evidence showed that the exercise pressor reflex played a key role in exaggerating the sympathetic and pressor responses to exercise in humans and animal models of peripheral artery disease [7, 8, 9, 10]. The afferent arm of this reflex is comprised of mechano‐ and metabosensitive Group III and IV afferent fibers [11, 12], whose endings originate in the interstitial space of contracting myocytes and their accompanying vessels and tendons [13, 14]. In rats with simulated peripheral artery disease, the responses of Group III and IV afferents to static contraction are significantly greater than those in control rats with freely perfused hindlimb muscles [15]. Thus, both the mechanical and metabolic components of the exercise pressor reflex are presumed to be exaggerated in this model [16, 17, 18].

The stimuli responsible for evoking the exaggerated metabolic component of the exercise pressor reflex in patients with peripheral artery disease are not fully determined, but several findings suggest that accumulation of cyclooxygenase by‐products [19, 20], ATP [18, 21], and acidic compounds such as lactate, inorganic phosphate, and hydrogen ions play a key role [17, 22]. Evidence from these experiments was primarily collected through injection of metabolic compounds and/or pharmacological blockade of their targeted receptors. Although very informative, these procedures always raise the possibility that off‐target effects mask the intended activation/inhibition. To further determine the role of a given metabolite in exaggerating the metabolic component of the exercise pressor in peripheral artery disease, a procedure that removes the targeted metabolite, for example, through genetic manipulation would be needed.

Recently, a genetically modified rat whose muscles did not produce hydrogen ions and lactate during static contractions under both freely perfused and ischemic conditions became available to us [23]. This model in combination with our decerebrated animal preparation [24] provided a unique opportunity to establish the role played by hydrogen and lactate ions in exaggerating the sympathetic and pressor responses to exercise in chronic ischemia. For example, we found that acute ischemia (~30 s) did not potentiate the exercise pressor reflex in the genetically modified knock out rats [23]. For this study, we hypothesized that the pressor response to static contraction is exaggerated in wild type rats with simulated peripheral artery disease (i.e., 72 h of femoral artery ligation). In contrast, we hypothesized that the effect of simulated peripheral artery disease on the pressor response to static contraction is reduced in knock‐out rats.

## 2 Methods

### 2.1 Ethical Approval

The Institutional Care and Use Committee of the Pennsylvania State University College of Medicine approved all of the procedures (IACUC:PRAM201647038). The authors understood and conformed to the ethical ARRIVE guidelines for animal use in research.

### 2.2 Animal Characteristics, Wellness, and Sample Size

Experiments were conducted at constant room air temperature (21°C). The rats were generated on a Wistar‐Kyoto breed by the Gene Editing Resource Center of the Medical College of Wisconsin. The *pygm* gene responsible for producing the myophosphorylase enzyme was edited with the use of CRISPER‐Cas 9 technology to produce a 767 amino acid deletion of the myophosphorylase protein so that skeletal muscle myocytes produced a non‐functional remnant comprised of 75 amino acids. Using 31P‐MRS and blood collection, we previously showed that during exercise, concentrations of intramuscular hydrogen ions and blood lactate increased in pygm^+/+^ rats but remained unchanged in pygm^−/−^ rats. Likewise, concentrations of intramuscular pH and blood lactate were exaggerated by exercise with acute muscle ischemia in pygm^+/+^ rats, whereas exercise with acute muscle ischemia had no effect on the concentration of these metabolites in pygm^−/−^ [23].

Adult *pygm* wildtypes (*pygm* ^+/+^), and *pygm* knockout (*pygm* ^−/−^) rats were used and were bred from *pygm* ^+/−^ parents. Male and female rats were used randomly. A total of 14 *pygm* ^+/+^ (7 males: 30 ± 2 weeks old, 434 ± 35 g; 7 females: 29 ± 3 weeks old, 263 ± 17 g) and 14 *pygm* ^−/−^ (7 males: 27 ± 6 weeks old, 436 ± 27 g; 7 females: 30 ± 5 weeks old, 277 ± 16 g) were used in our experiments. The rats were housed within the central animal facility of the Pennsylvania State College of Medicine, where they had access to food and water ad libitum, and a 50:50 light/dark cycle. All attempts were made to minimize animal discomfort and pain. The experimenter was blinded as to whether the rat was either *pygm* ^+/+^ or *pygm* ^−/−^.

### 2.3 Experimental Protocols in vivo

Peripheral artery disease was simulated by ligating the right or left femoral artery 72 h before conducting the experiments (surgical procedures detailed below) [10]. Three maneuvers were performed on the triceps surae muscles of each rat. In the first maneuver, the exercise pressor reflex was evoked by statically contracting the triceps surae muscles. In the second maneuver, the responsiveness of the mechanical component of the exercise pressor reflex (i.e., the muscle mechanoreflex) was evaluated by stretching the triceps surae muscles [25]. This maneuver is believed to mimic the mechanical stimulation of the muscles without producing intramuscular metabolites. In the third maneuver, the responsiveness of the metabolic component of the exercise pressor reflex (i.e., the metaboreflex) was evaluated by injecting lactic acid (0.1 mL, 24 mM, pH 2.4) or diprotonated phosphate (0.1 mL, 24 mM, pH 6.0) solutions into the arterial circulation of the triceps surae muscles. Lactic acid and diprotonated phosphate are two metabolites that are produced by contracting muscles [26] and are potent activators of Group III and IV afferents [27, 28]. All maneuvers were separated by at least 10 min of recovery and were performed on the leg with simulated peripheral artery disease and on the contralateral leg with intact arterial circulation. The contralateral leg was used as a control condition for each rat. Compared to the conventional approach that uses different rats for control and experimental conditions, our procedure improves our experimental design by eliminating inter‐animal biological variability, variability induced by slight differences between surgeries, enabling paired statistical comparison, improving statistical power and reducing the number of rats used.

#### 2.3.1 Surgical Procedure for Ligating the Femoral Artery

Seventy‐two hours before the experiment, we ligated the left or right femoral artery using a 6.0 silk suture. The rat was anesthetized by inhalation of 4% isoflurane in oxygen. The femoral artery was isolated from its vein and nerve and was ligated approximately 5 mm below the inguinal ligament. The wound was sutured, and 1–2 mg/kg of bupivacaine was injected subcutaneously. This surgical procedure simulated the blood flow pattern to skeletal muscle seen in peripheral artery disease. Specifically, muscle metabolic demand is met at rest but not during exercise. Likewise, femoral artery ligation decreases maximal blood flow to ~10%–20% of normal [29, 30].

#### 2.3.2 Surgical Procedures for Reflex Experiments

Each rat was anesthetized initially by inhalation of 4% isoflurane in O_2_. The rat's trachea was cannulated, and its lungs were mechanically ventilated (model 683; Harvard Apparatus Inc., Holliston, MA). The concentration of isoflurane was reduced to 2% for the rest of the surgery. The left and right common carotid arteries and right jugular vein were cannulated (RPT040; Braintree Scientific Inc., Braintree, MA) to record arterial blood pressure (P23XL; Gould‐Statham Instruments Inc., Los Angeles, CA), draw arterial blood samples, and inject drugs, respectively. We cannulated the left and right superficial epigastric arteries (SUBL‐140; Braintree Scientific Inc., Braintree, MA, USA), which are side branches of the femoral artery, to inject drugs into the circulation of the triceps surae muscles. A snare (2.0 silk suture) was placed around the left and right femoral arteries and veins ~0.5 cm upstream to the superficial epigastric artery and vein to trap the injected solution in the hindlimb circulation.

The head of the rat was secured in a Kopf stereotaxic unit. The hip and ankles were secured with metal clamps to prevent movement during the contraction or stretch procedures. The calcaneus bones were severed, and their Achilles tendons were connected to a force transducer (FT10; Grass Instrument Co., Quincy, MA) and a rack and pinion device. The left and right tibial nerves were isolated and hooked with a bipolar stainless‐steel electrode. Using a blunted spatula, we decerebrated the rat by sectioning and removing all neural tissue ~1 mm rostral to the superior colliculus [31, 32]. Isoflurane was discontinued, and the lungs were ventilated with room air. Using a retroperitoneal approach, a branch of the left renal nerve was dissected, glued to a bipolar platinum–iridium electrode (778000; A‐M Systems) and connected to a high‐impedance probe (HIP511; Grass Instrument Co., Quincy, MA) to record sympathetic renal nerve activity [33]. Blood arterial PO_2_ (100–150 mmHg), PCO_2_ (35–40 mmHg), and [HCO_3_^−^] (22–26 mmol L^−1^) were kept within physiological range. Body temperature was maintained around 37°C using a heating lamp. At the end of the experiment, hexamethonium bromide (0.5 mL; 20 mg mL^−1^; Sigma‐Aldrich) was injected intravenously to verify that the recorded nerve activity corresponded to post‐ganglionic sympathetic activity and to quantify background noise [32]. Data were discarded in the event that hexamethonium had no effect on the recorded signal (*n* = 4). At the end of the experiment, the decerebrated rats were euthanized by intravenous injection of a supersaturated KCl solution and the chest was opened.

#### 2.3.3 Contraction of the Triceps Surae Muscles to Evoke the Exercise Pressor Reflex

All contractions were conducted isometrically. Baseline tension of the triceps surae muscles was set at 60–100 g. Motor threshold was determined by applying single pulses (0.01 ms) to the left or right tibial nerve to find the minimal current needed to evoke a muscle twitch (S88; Grass Instrument Co., Quincy, MA). The triceps surae muscles were then statically contracted for 30 s by stimulating the tibial nerve (40 Hz; ~1.5 times motor threshold). The side that was investigated first was randomly selected. After 10 min of recovery, the triceps surae muscles from the opposite side were contracted using the same procedure.

At the end of the experiment, the decerebrated rat was paralyzed by intravenous injection of pancuronium bromide (1 mg/mL, 200 μL) after which the tibial nerves were stimulated for 30 s at 40 Hz with the highest current used to evoke their respective contractions. If an increase in blood pressure was observed, the data obtained from the contraction experiment was excluded (*n* = 1).

#### 2.3.4 Stretch of the Triceps Surae Muscles to Evoke the Mechanoreflex

Following the contraction experiments, we measured the pressor response to passive stretch [25]. To avoid potential muscle contractions during the stretch procedure, the rats were paralyzed by intravenous injection of pancuronium bromide (1 mg/mL, 200 μL). The left or right triceps surae muscles were stretched for 30 s by turning a rack and pinion attached to the Achilles tendon until a tension of ~700 g was reached. The side that was investigated first was randomly selected. After 10 min of recovery, the other triceps surae muscles were stretched using the same procedure.

#### 2.3.5 Injection of Acidic Solutions to Evoke the Metaboreflex

Following the stretch experiments, we injected a solution of lactic acid (24 mM, 0.1 mL, pH = 2.6) or a solution of diprotonated phosphate (24 mM, 0.1 mL, pH = 6.0) into the left or right superficial epigastric arteries. The solutions were flushed with normal saline (0.1 mL) to remove all active compound in the catheter. Each injection was separated by an interval of at least 10 min. The side that was investigated first was randomly selected. To determine that injections into the hindlimb arterial circulation accessed the triceps surae muscles, we injected 0.2 mL of Evans Blue dye into the superficial epigastric artery catheter of each rat tested. We considered that the injected solution circulated to the triceps surae muscles if they turned blue. If the color of the muscles did not change, we excluded the data from the study (*n* = 2).

#### 2.3.6 Data Analysis for In Vivo Experiments

Renal sympathetic nerve activity was amplified (gain: ×10,000) and filtered (bandpass 100 Hz–1 kHz) with a Grass P511 pre‐amplifier (Grass Instrument Co., Quincy, MA). Tension and arterial blood pressure signals were amplified using Gould Universal amplifiers (Gould‐Statham Instruments Inc., CA). Except renal sympathetic nerve activity, which was recorded at 10 kHz, all signals were recorded at 1 kHz using an A/D converter (Micro1401 mkII; Cambridge Electronic Design Limited, Cambridge, UK) and its associated commercially available software (Spike2, 7.20, RRID: SCR_000903; Cambridge Electronic Design Limited). Heart rate was calculated beat by beat from the arterial pressure pulse signal and expressed as beats per minute. The renal sympathetic nerve activity signal was rectified.

To determine the effects of the maneuvers, we calculated the peak and integrated response of each variable. The latter was calculated by integrating the area under the curve during the 30 s contraction or stretch period and then subtracting from this value the area under the curve measured during the immediately preceding 30 s baseline period. The integration of the response provides a measure of the entire response to contraction or stretch, unlike the peak value, which represents the instantaneous maximal value of the response. The time course of blood pressure, heart rate, tension, and renal sympathetic nerve activity to static contraction or passive stretch was plotted by averaging the mean signal every 2 s.

### 2.4 Western Blot Analysis of EP4 Expression in Dorsal Root Ganglia

Two studies showed that 72 h of femoral artery ligation resulted in upregulation of EP4 receptors and contributed to exaggerate the exercise pressor reflex [19, 20]. As a control, we verified that the effects of our model of myophosphorylase knock‐out on the exercise pressor reflex did not prevent the EP4 upregulation which is induced by ischemia, we measured expression of this receptor in dorsal root ganglia. In this set of experiments, three pygm^+/+^ and three pygm^−/−^ rats had their left femoral artery ligated. The contralateral side of each animal served as the sham control (i.e., freely perfused). Seventy‐two hours post‐ligation, the animals were sacrificed and the L_4_–L_6_ dorsal root ganglion tissues from the freely perfused and ligated sides were collected. The protein from each tissue was isolated as previously described [20]. Briefly, the protein from each dorsal root ganglion tissue was prepared with the Nucleospin RNA/Protein Kit (Macherey‐Nagel) according to the manufacturer's instructions, and the concentration was measured with the Qubit 2.0 Fluorometer the Qubit Protein Assay Kit (both from Thermo‐Fisher). The “Wes” Simple Western system with the 66–440 kDa Simple Western Separation Module (Protein Simple) was employed for EP4 detection. The protein samples were loaded onto the plate at 0.75 μg/lane and EP4 receptor expression was detected employing a mouse monoclonal anti‐EP4 primary antibody (Santa Cruz Biotechnology, cat. # sc‐55 596), diluted to 1:25, and a horseradish peroxidase‐conjugated anti‐mouse secondary antibody provided in the kit (Protein Simple). The EP4 receptor chemiluminescence peaks were identified in the range of 45–55 kDa and their size was measured by the Wes System software through the integration of a fitted Gaussian function. Afterwards, the peak areas for each sample were normalized across lanes based on the measurement of total protein in each sample using the accessory kit (DM‐TP01; Protein Simple).

### 2.5 Drug Preparations

Lactic acid (Sigma‐Aldrich; 24 mM; pH 2.66) and diprotonated phosphate (24 mM; pH 6.0) solutions were dissolved in 0.9% saline and stored at +4°C. Diprotonated phosphate solution was made by mixing 24 mM of Na_2_HPO_4_ with 24 mM of NaH_2_PO_4_ in 10 mM HEPES [4‐(2‐hydroxyethyl)‐1‐piperazineethanesulfonic acid]. The pH of the stock solution was decreased to 6.0 by adding HCl and, if necessary, adjusted on a weekly basis.

### 2.6 Statistical Analysis

Data are presented as means ± SD. Using a Shapiro–Wilk test, we determined whether our samples followed a normal distribution or not. The pre‐ to post‐stimulus (i.e., static contraction, passive stretch or chemical injection) change in peak, or integrated responses (i.e., blood pressure, tension, blood flow, sympathetic activity) were evaluated using a two tailed Student's paired *t*‐test or its non‐parametric equivalent Wilcoxon's test. The effects of sex on the responses to the different maneuvers were evaluated using a two tailed Student's unpaired *t*‐test or its non‐parametric equivalent Mann–Whitney's test. The effects of femoral artery ligation on the ∆ change of our variables was evaluated with a two‐way ANOVA with one between (*type effect: pygm* ^*+/+*^ vs *pygm* ^*−/−*^), and one within effect (*Ligation effect: freely perfused* vs *72 h ligated femoral artery*). Differences in the time course of the variables were evaluated using three‐way ANOVA with a one between (*type effect*), and a two within effects (*Ligation and time effect*). Given that no difference between males and females was found in most of our variables (see Section 3), males and females were pooled together. The criterion for statistical significance was *p* < 0.05. When a statistical difference was found with the two‐way ANOVA, post hoc multiple‐comparison analysis was performed using Tukey's honestly significant difference test. When a statistical difference was found with the three‐way ANOVA, post hoc multiple‐comparison with Holm‐Bonferroni correction analysis was performed only on the comparisons of interest (pygm^+/+^ or pygm^−/−^ freely perfused vs. ligation at a corresponding time point). This statistical plan was designed a priori and optimizes the correction factors for multiple comparisons to limit the risk of type II error while limiting the risk of type I error [34, 35]. Effect size was calculated using partial *η* ^2^ (_*p*_ *η* ^2^) for ANOVAs and Cohen's *d* for the post hoc comparisons. A Cohen's *d* index for effect size was considered as small, medium, or large when *d* was close to 0.2, 0.5, or 0.8, respectively [36]. A _*p*_ *η* ^2^ for effect size was considered as small, medium, or large when _*p*_ *η* ^2^ was close to 0.02, 0.13, or 0.26, respectively [36]. Statistical analyses were conducted using Prism (RRID: SCR_002798; 10.3.0; GraphPad software; Boston, MA, USA).

## 3 Results

### 3.1 Baseline Variables

No difference in baseline blood pressure (*p* > 0.101, *d* < 0.525), heart rate (*p* > 0.292, *d* < 0.425), and renal sympathetic nerve activity (*p* > 0.425, *d* < 0.340) was found between pygm^+/+^ and pygm^−/−^ rats. Static contraction, passive stretch, and injection of lactic acid and diprotonated phosphate significantly elevated blood pressure (*p* < 0.0003, *d* > 1.40), heart rate (*p* < 0.0069, *d* > 0.903), and renal sympathetic nerve activity (Table 1; *p* < 0.0142, *d* > 0.959). Except for pygm^−/−^ rats, which showed lower peak heart rate (*p* < 0.0283, *d* > 1.33) and renal sympathetic nerve activity (*p* < 0.0192, *d* > 1.45) responses to passive stretch from both limbs in females compared to males, no difference between males and females was found in the blood pressure (*p* > 0.215, *d* < 0.716), heart rate (*p* > 0.135, *d* < 0.858), and sympathetic activity responses (*p* > 0.257, *d* < 0.709) to our different maneuvers.

**TABLE 1:** Baseline values for blood pressure, heart rate, tension, and renal sympathetic nerve activity during the different experiments.

Variable	Type	Static contraction			Passive stretch			Lactic acid			Diprotonated phosphate		
		*n*	FP	Lig	*n*	FP	Lig	*n*	FP	Lig	*n*	FP	Lig
Blood pressure (mmHg)	pygm^+/+^	14	75 ± 12	69 ± 7	14	86 ± 16	85 ± 15	13	97 ± 18	93 ± 15	13	94 ± 11	92 ± 12
	pygm^−/−^	13	67 ± 14	70 ± 15	14	78 ± 14	72 ± 13	13	87 ± 19	89 ± 15	13	82 ± 17	81 ± 16
Heart rate (beats min^−1^)	pygm^+/+^	14	275 ± 39	263 ± 44	14	297 ± 57	292 ± 58	13	315 ± 52	316 ± 52	13	306 ± 58	308 ± 58
	pygm^−/−^	13	274 ± 31	277 ± 25	14	312 ± 47	309 ± 47	13	329 ± 40	328 ± 41	13	328 ± 42	329 ± 41
RSNA (μV)	pygm^+/+^	13	1.41 ± 0.72	1.37 ± 0.77	13	1.50 ± 0.82	1.38 ± 0.82	12	1.44 ± 0.46	1.48 ± 0.5	12	1.5 ± 0.57	1.56 ± 0.63
	pygm^−/−^	10	1.27 ± 0.86	1.29 ± 0.85	11	1.51 ± 0.96	1.36 ± 0.84	10	1.87 ± 1.05	1.79 ± 1.05	10	1.95 ± 1.12	1.85 ± 1.06
Tension (g)	pygm^+/+^	14	74 ± 14	75 ± 12	14	84 ± 11	86 ± 12	NA	NA	NA	NA	NA	NA
	pygm^−/−^	13	83 ± 15	73 ± 12	14	97 ± 19	87 ± 10	NA	NA	NA	NA	NA	NA

*Note:* Data are presented as mean ± SD.

Abbreviations: FP, experiments from hindlimb muscles with freely perfused circulation; Lig, experiments from hindlimb muscles with 72 h femoral artery ligated; NA, not applicable; pygm^−/−^, animals with knock‐out pygm gene; pygm^+/+^, animals with wild‐type pygm gene; RSNA, renal sympathetic nerve activity.

### 3.2 Effects of Chronic Femoral Artery Ligation on the Exercise Pressor Reflex in Rats With or Without Intact pygm Gene

**FIGURE 1:**
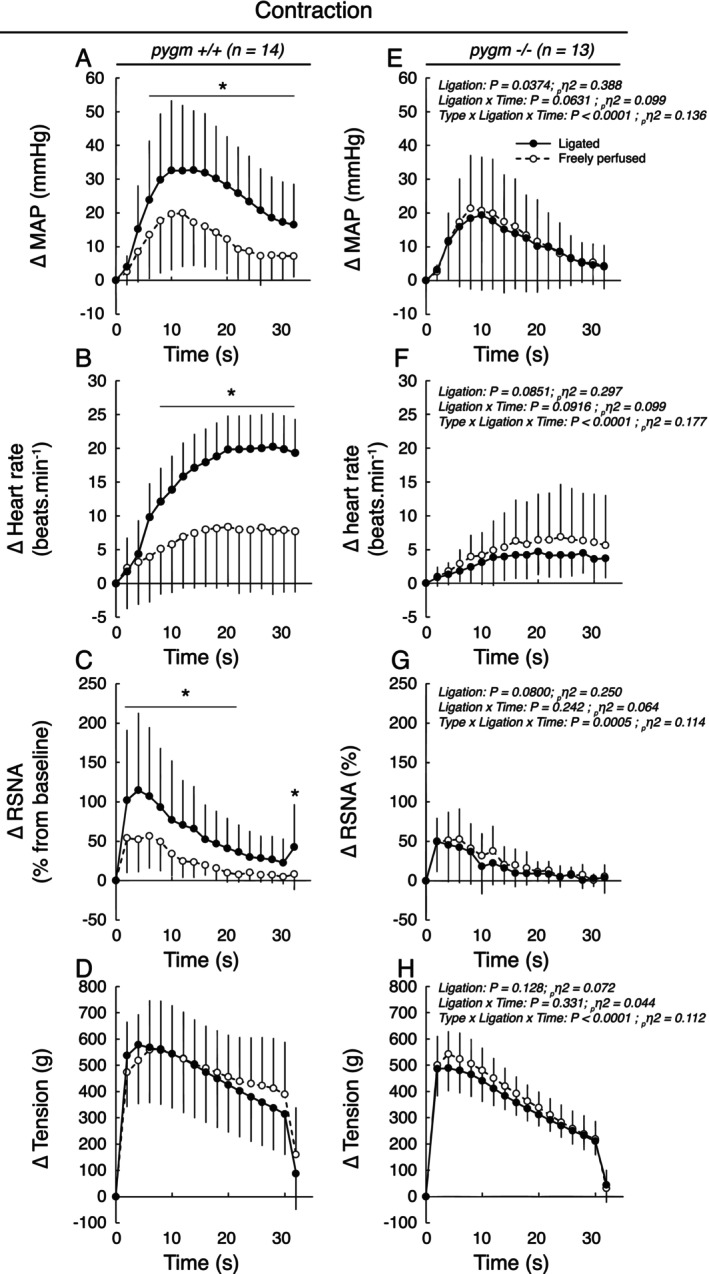
Effects of static contraction with or without chronic femoral artery ligation on the pressor, cardioaccelerator, sympathetic, and tension responses in pygm^+/+^ and pygm^−/−^ rats. Data are presented as the mean ± SD changes over time in blood pressure (A, E), heart rate (B, F), RSNA (C, G) and tension (D, H) induced by static contraction. Males and females were pooled in both groups. The averaged time course includes 2 s of baseline, 30 s of contraction and 2 s after the end of contraction. Data were analyzed using three‐way ANOVA with repeated measures. MAP, mean arterial pressure; pygm^+/+^, rats with wild‐type pygm gene (*n* = 14; 7 females); pygm^−/−^, rats with knock‐out pygm gene (*n* = 13; 6 females); RSNA, renal sympathetic nerve activity; *p < 0.05 between freely perfused vs. ligated hindlimb circulation at corresponding time points.

**FIGURE 2:**
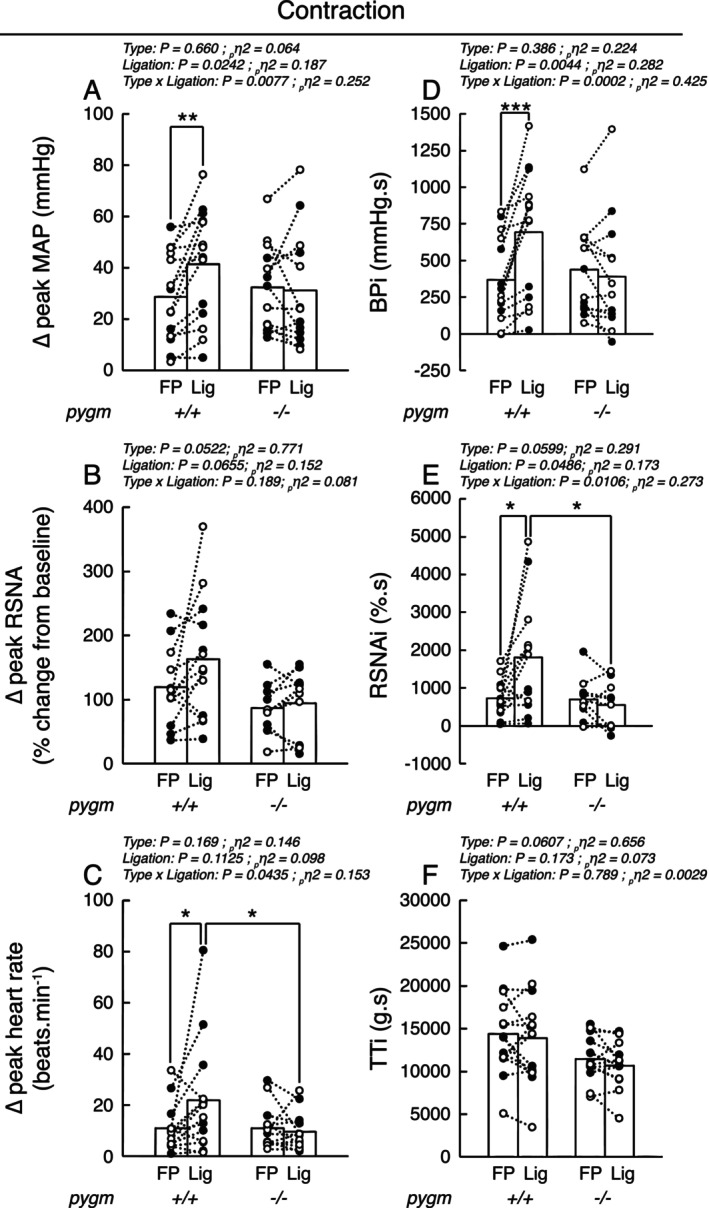
Effect of static contraction with or without chronic femoral artery ligation on the peak or integrated pressor, cardioaccelerator, sympathetic and tension responses in pygm^+/+^ and pygm^−/−^ rats. Data are presented as individual (open/black dots) and group means (open bars) for the peak and integrated changes in blood pressure (A, D), renal sympathetic nerve activity (B, E), peak change in heart rate (C) and integrated tension (F) evoked by static contraction. Black dots represent males, white dots represent females. Data were analyzed using two‐way ANOVA with repeated measures. BPi, blood pressure index calculated from the integrated blood pressor response to contraction; FP, response from freely perfused muscles; Lig, response from chronic femoral artery ligated muscles; MAP, mean arterial pressure; pygm^−/−^, rats with knock‐out pygm gene (*n* = 13; 6 females); pygm^+/+^, rats with wild‐type pygm gene (*n* = 14; 7 females); RSNA, renal sympathetic nerve activity; RSNAi, integrated RSNA response; TTi, tension time index calculated from the integrated tension response to contraction; *p < 0.05 between corresponding datasets; **p < 0.01; ***p < 0.001.

Static contraction of the triceps surae muscles whose femoral artery was freely perfused increased blood pressure (*p* < 0.0001, *d* > 1.65), heart rate (*p* < 0.0007, *d* > 1.19), and renal sympathetic nerve activity (*p* < 0.0020, *d* > 1.37) in both groups (Figures 1 and 2). These responses were not different in pygm^−/−^ rats compared to those in pygm^+/+^ rats (Figures 1 and 2; *p* > 0.454, *d* < 0.056). Following 72 h (i.e., chronic) of femoral artery ligation, the pressor, cardioaccelerator, and renal sympathetic responses to static contraction were exaggerated in pygm^+/+^ rats (Figures 1A and 2A,D). In contrast, chronic femoral artery ligation had no effect on these responses in pygm^−/−^ rats (Figures 1D and 2A,D; *p* > 0.671, *d* > 0.143). Specifically, in pygm^+/+^ rats, chronic femoral artery ligation increased the peak and integrated pressor responses to static contraction by an average of 45% (*p* = 0.0016, *d* = 0.656) and 88% (*p* < 0.0001, *d* = 0.881) compared to the responses evoked from their contralateral, freely perfused, triceps surae muscles (Figure 2A,D). This was associated with a 100% larger cardioaccelerator response (*p* = 0.0234, *d* = 0.656; Figures 1B and 2C) and with a 37% (*p* = 0.0234, *d* = 0.563) and 147% (*p* = 0.0026, *d* = 0.958) stronger peak and integrated renal sympathetic nerve activity (Figure 2B,E). Analysis of the time course of blood pressure, heart rate, and renal sympathetic nerve activity revealed that the responses of these variables when the femoral artery was ligated differed from the contralateral responses with a latency of respectively 6, 8, and 2 s and remained exaggerated for the rest of the maneuver (Figure 1). Importantly, no difference in the peak or integrated tension responses to static contraction was found between groups or between hindlimbs with or without chronic femoral artery ligation (Figures 1D,H and 2F).

### 3.3 Effects of Chronic Femoral Artery Ligation on the Mechanoreflex Evoked in Rats With or Without Intact pygm Gene

**FIGURE 3:**
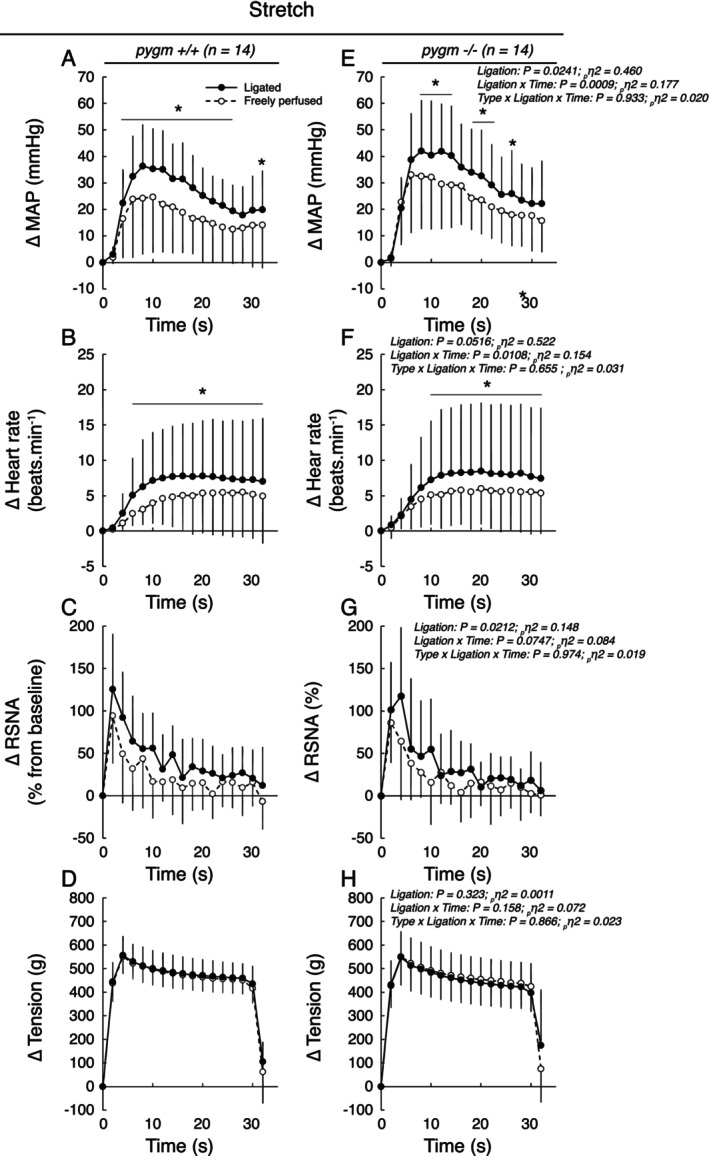
Effects of passive stretch with or without chronic femoral artery ligation on the pressor, cardioaccelerator, sympathetic, and tension responses in pygm^+/+^ and pygm^−/−^ rats. Data are presented as the mean ± SD changes over time in blood pressure (A, E), heart rate (B, F), RSNA (C, G) and tension (D, H) induced by passive stretch. Males and females were pooled in both groups. The averaged time course includes 2 s of baseline, 30 s of contraction and 2 s after the end of stretch. Data were analyzed using three‐way ANOVA with repeated measures. MAP, mean arterial pressure; pygm^−/−^, rats with knock‐out pygm gene (*n* = 14; 7 females); pygm^+/+^, rats with wild‐type pygm gene (*n* = 14; 7 females); RSNA, renal sympathetic nerve activity; *p < 0.05 between freely perfused vs. ligated hindlimb circulation at corresponding time points.

**FIGURE 4:**
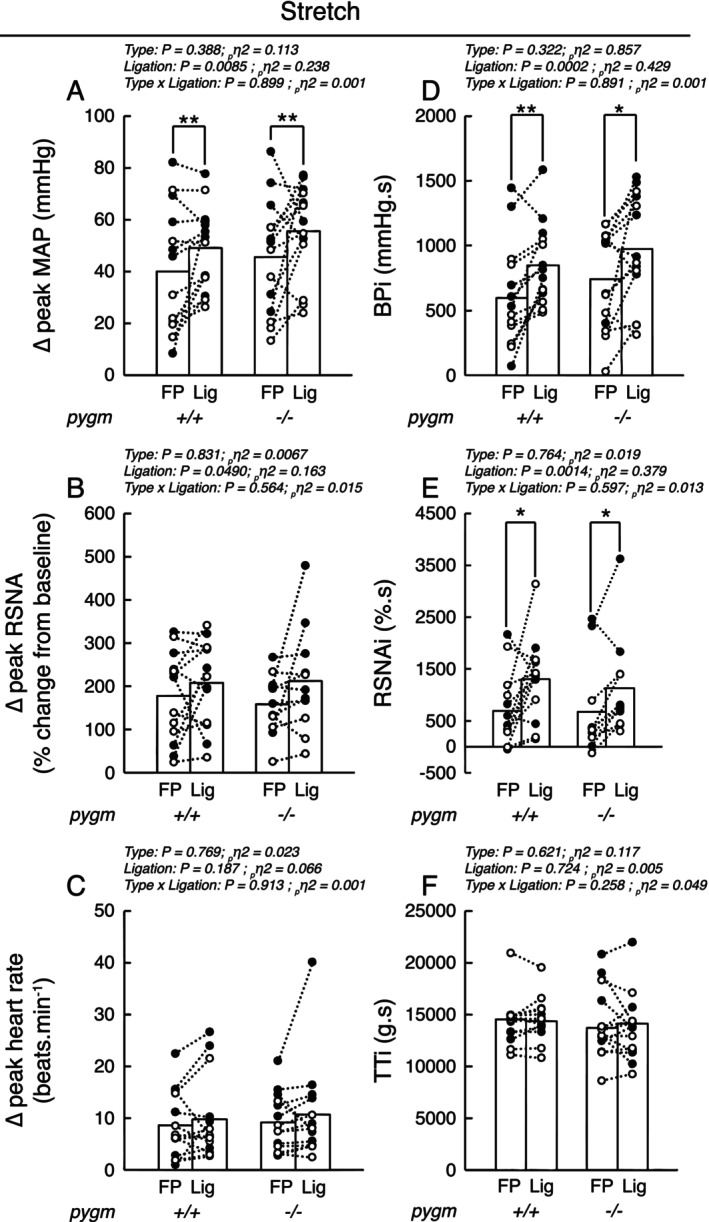
Effect of passive stretch with or without chronic femoral artery ligation on the peak or integrated pressor, cardioaccelerator, sympathetic, and tension responses in pygm^+/+^ and pygm^−/−^ rats. Data are presented as individual (open/black dots) and group means (open bars) for the peak and integrated changes in blood pressure (A, D), renal sympathetic nerve activity (B, E), peak change in heart rate (C), and integrated tension (F) evoked by passive stretch. Black dots represent males, white dots represent females. Data were analyzed using two‐way ANOVA with repeated measures. BPi, blood pressure index calculated from the integrated blood pressor response to stretch; FP, response from freely perfused muscles; Lig, response from chronic femoral artery ligated muscles; MAP, mean arterial pressure; pygm^−/−^, rats with knock‐out pygm gene (*n* = 14; 7 females); pygm^+/+^, rats with wild‐type pygm gene (*n* = 14; 7 females); RSNA, renal sympathetic nerve activity; RSNAi, integrated RSNA response; TTi, tension time index calculated from the integrated tension response to stretch; *p < 0.05 between corresponding datasets; **p < 0.01.

Passive stretch of the triceps surae muscles whose femoral arteries were freely perfused increased blood pressure (*p* < 0.0001, *d* > 1.64), heart rate (*p* < 0.0002, *d* > 1.33) and renal sympathetic nerve activity (*p* < 0.0002, *d* > 1.60) in both groups. These responses were not different in pygm^−/−^ rats compared to pygm^+/+^ rats (Figures 3 and 4). In both groups, 72 h of chronic femoral artery ligation exaggerated the pressor, heart rate and renal sympathetic responses to passive stretch (Figures 3 and 4). The magnitude by which these responses were exaggerated was not different in pygm^−/−^ rats compared to that in pygm^+/+^ rats. Specifically, in pygm^+/+^ rats, the peak and integrated pressor responses to passive stretch from the triceps surae muscles with chronic femoral artery ligation were increased in average by 23% (*p* = 0.0089, *d* = 0.443) and 42% (*p* = 0.0068, *d* = 0.691) compared to the contralateral, freely perfused, triceps surae muscles (Figure 4A,D). Likewise, in pygm^−/−^ rats, these variables were exaggerated by a comparable 22% (*p* = 0.0089, *d* = 0.495) and 32% (*p* = 0.0109, *d* = 0.583). In addition, the integrated renal sympathetic nerve activity was exaggerated in both groups of rats by 88% (*p* = 0.0490, *d* = 0.413) and 67% (*p* = 0.0014, *d* = 0.659; Figure 4B,E). No difference in the time‐course of blood pressure, heart rate and renal sympathetic nerve activity in response to passive stretch was found between groups. The latency by which blood pressure and heart rate were exaggerated was comprised between 4 and 10 s (Figure 3A,E). No difference in the peak or integrated tension responses to passive stretch was found between groups or between passive stretch with or without chronic femoral artery ligation (Figures 3D,H and 4F).

### 3.4 Effects of Chronic Femoral Artery Ligation on the Responses to Lactic Acid and Diprotonated Phosphate Injections in Rats With or Without Intact pygm Gene

**FIGURE 5:**
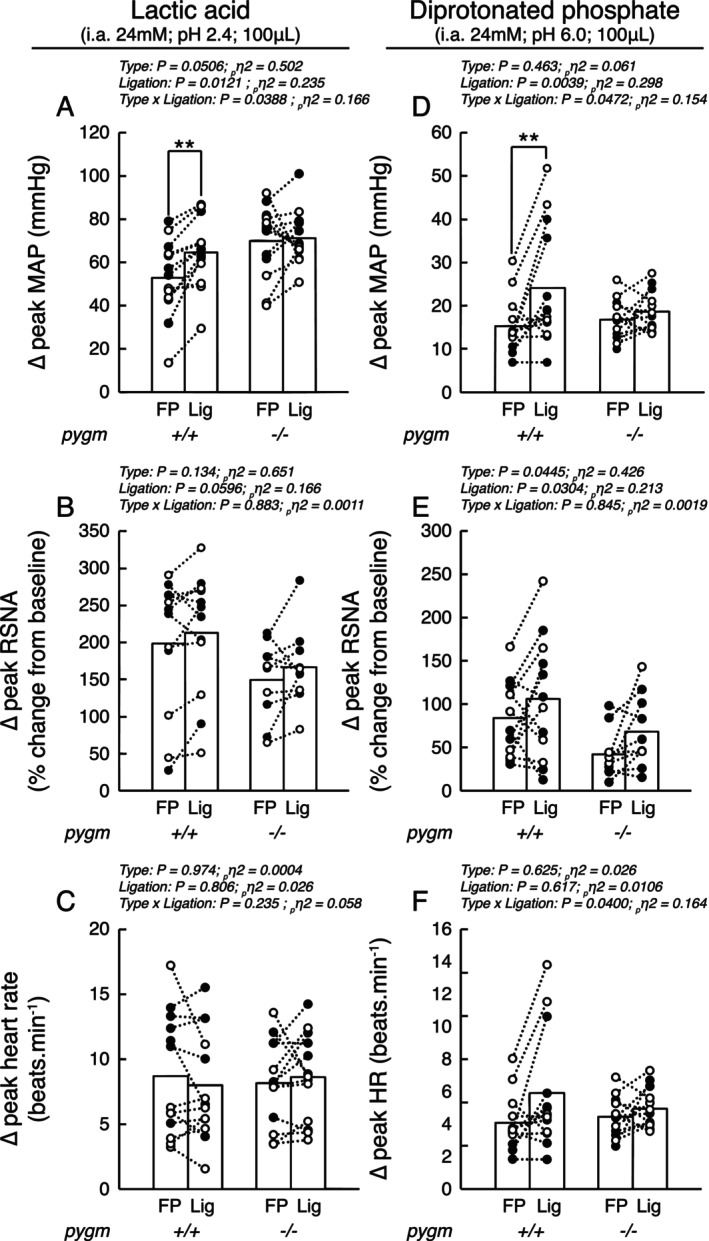
Effect of injecting lactic acid or diprotonated solution into the arterial circulation of the triceps surae muscles with or without chronic femoral artery ligation in pygm^+/+^ and pygm^−/−^ rats. Data are presented as individual (open/black dots) and group means (open bars) for the peak changes in blood pressure (A, D), renal sympathetic nerve activity (B, E), and heart rate (C, F) following injection of lactic acid (A–C) or deprotonated phosphate (D–F). Black dots represent males, white dots represent females. Data were analyzed using two‐way ANOVA with repeated measures. FP, response from freely perfused muscles; Lig, response from chronic femoral artery ligated muscles; MAP, mean arterial pressure; pygm^−/−^, rats with knock‐out pygm gene (*n* = 13; 6 females); pygm^+/+^, rats with wild‐type pygm gene (*n* = 13; 6 females); RSNA, renal sympathetic nerve activity; *p < 0.05 between corresponding datasets; **p < 0.01.

Injecting lactic acid or diprotonated phosphate into the circulation of the triceps surae muscles whose femoral arteries were freely perfused increased blood pressure (*p* < 0.0001, *d* > 2.37), heart rate (*p* < 0.0069, *d* > 0.903), and renal sympathetic nerve activity (*p* < 0.0142, *d* > 0.959) in both groups (Table 1; Figure 5). These responses were not different in pygm^−/−^ compared to pygm^+/+^ (Figure 5). When the solutions were injected into the hindlimb circulation with chronic femoral artery ligation, the pressor response to injection of lactic acid and diprotonated phosphate solutions was increased by 22% (*p* = 0.0040, *d* = 0.694) and 58% (*p* = 0.0020, *d* = 0.817) in pygm^+/+^ rats (Figure 5A,D). No difference was found in pygm^−/−^ rats between the procedures (*p* > 0.690, *d* < 0.402). In both groups, chronic femoral artery ligation had no effect on either heart rate (*p* > 0.136, *d* < 0.647; Figure 5C,F) or renal sympathetic nerve activity (*p* > 0.195, *d* < 0.738; Figure 5B,E).

### 3.5 Effects of Chronic Femoral Artery Ligation on the Protein Expression of EP4 Receptors in Rats With or Without Intact Pygm Gene

**FIGURE 6:**
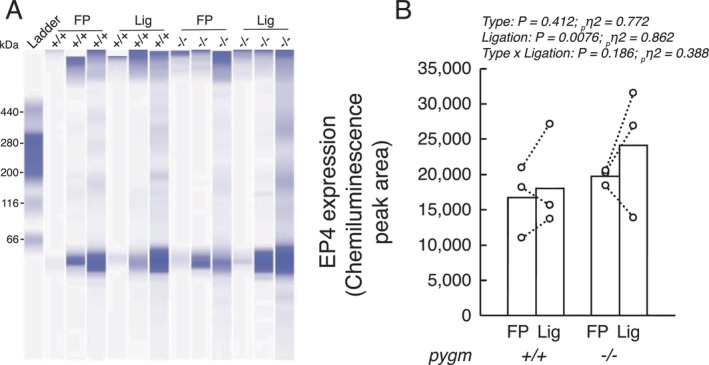
Endoperoxide 4 (EP4) receptor expression in dorsal root ganglia isolated from pygm^+/+^ and pygm^−/−^ rats with freely perfused or chronically ligated femoral artery. Full, unedited, Western blot (A) and quantification (B) of endoperoxide 4 (EP4) receptors in dorsal root ganglia (L_4_–L_6_). Data were analyzed using two‐way ANOVA with repeated measures. FP represents dorsal root ganglia tissue from rats with freely perfused circulation; Lig represents dorsal root ganglia tissue obtained from rats with ligated femoral artery for 72 h; pygm^+/+^, rats with wild‐type pygm gene (*n* = 3); pygm^−/−^, rats with knock‐out pygm gene (*n* = 3).

No type or interaction effect was found on the expression levels of EP4 receptors in lumbar dorsal root ganglia of pygm^+/+^ and pygm^−/−^ rats (Figure 6). However, a main ligation effect was found. This shows that chronic femoral artery ligation contributed to increase the expression of EP4 receptors regardless of the genetic profile of the rats.

## 4 Discussion

We identified a crucial mechanistic role played by hydrogen and lactate ions in exaggerating the exercise pressor reflex in a rat model of peripheral artery disease. Using a genetic model of knock out rats in which myophosphorylase was rendered non‐functional, we found that the exercise pressor reflex was not exaggerated in pygm^−/−^ rats with simulated peripheral artery disease whereas it was nearly doubled in pygm^+/+^ rats. The lack of functional myophosphorylase enzyme blunts the production of intramuscular hydrogen ions and lactate, directly linking these metabolites to the exaggerated blood pressure, heart rate, and sympathetic responses documented in peripheral artery disease. The strikingly similar cardiovascular and sympathetic responses to static contraction in pygm^−/−^ rats with or without chronically occluded femoral arteries echo our previous results showing similar effects of myophosphorylase knockout in the pressor response evoked by acute occlusion of the femoral artery (~30 s vs. 72 h) [23]. The present findings provide strong evidence that muscle acidosis and lactate production are paramount contributors to the exaggerated exercise pressor reflex seen in peripheral artery disease.

### 4.1 Muscle Lactate and Acidosis Are Required to Exaggerate the Exercise Pressor Reflex in Peripheral Artery Disease

In line with numerous findings, chronic ligation of the femoral artery dramatically exaggerated blood pressure, heart rate and sympathetic responses to static contraction in rats with an intact *pygm* gene [10, 17, 18, 24, 37]. Uncertainty existed on the intramuscular metabolites that contributed to this exaggerated exercise pressor reflex in peripheral artery disease [38, 39]. Several authors suggested that ATP, prostaglandins, bradykinin, lactate and hydrogen ions, alone or in combination, were the key mediators [17, 18, 20, 37, 40, 41]. Our results showing that exaggeration of the exercise pressor reflex is abolished in pygm^−/−^ rats with simulated peripheral artery disease demonstrate that hydrogen ions and lactate are of major importance (Figures 1A and 2A,D). Importantly, the concentration of these two metabolites dramatically increases during ischemic contractions in pygm^+/+^ but not in pygm^−/−^ [23]. The lack of accumulation of protons and lactate in our knockout model of myophosphorylase [23] likely prevented the opening of a family of receptors localized on the nerve endings of Group III–IV afferent fibers, directly activated or sensitized by acidosis and lactate. Among others, ASIC3, but not ASIC1a [24] or TRPV1 [10, 42, 43], appears to be a good candidate. Unlike acute ischemia, which is not expected to alter the expression of metabosensitive channels expressed on the membrane of dorsal root ganglia, multiple studies showed that the ASIC3 channel is upregulated in the dorsal root ganglia of rats with either simulated peripheral artery disease or ischemia–reperfusion [44, 45, 46]. In addition, pharmacological blockade or genetic deletion of the ASIC3 channel has been shown to restore the exercise pressor reflex to normal values in these models [17, 22, 46]. In contrast, inhibiting ASIC1a or TRPV1 with pharmacological blockade [10, 24, 43] or genetic knock‐out [42] did not alter the exaggerated exercise pressor reflex in rats with acute or chronic ischemia.

The fact that preventing the intramuscular accumulation of hydrogen ions and lactate abolished the exaggerated exercise pressor reflex in pygm^−/−^ rats, rather than just reducing it, was surprising. Several authors showed that receptors such as purinergic 2X [18, 37], activated by ATP, bradykinin 2 [40, 41], activated by bradykinin, and endoperoxide 4 [19, 20], activated by prostaglandin E2, were involved in the augmented exercise pressor reflex induced by peripheral artery disease. One could have hypothesized that only a fraction of the exaggerated pressor response would have been removed by the removal of lactate and hydrogen ions. This view is coherent with the assumption that the molecular mechanisms responsible for evoking the exercise pressor reflex are compartmentalized, namely that each metabolite activates its associated receptor and contributes to a fraction of the response. In contrast, our data support the hypothesis that metabolites and their receptors act synergistically [47], with hydrogen ions being the initiating stimulus. In that context, removing the initiator would completely abolish the exaggerated exercise pressor reflex rather than a fraction of it. Support for this hypothesis is given by data showing that ATP [48, 49], prostaglandin E2 [50], and bradykinin [51] increased the sensitivity of ASIC3 channels, an effect caused by the activation of purinergic 2X and endoperoxide 4 receptors.

We speculate that hydrogen rather than lactate ions initiated the exaggerated exercise pressor reflex seen in peripheral artery disease because (1) hydrogen ion is the direct activator of ASICs whereas lactate is not [52]; (2) lactate has been shown to be a Ca^2+^ chelator for ASIC3; that is it removes a Ca^2+^ plug and facilitates the binding of hydrogen ions to ASIC3 [53, 54]; (3) injecting a solution of lactate at neutral pH does not produce a pressor response whereas injecting an acidic solution with no lactate does produce a pressor response [55].

### 4.2 Peripheral Artery Disease Exaggerates the Responsiveness of the Metabolic Component of the Exercise Pressor Reflex in pygm^+/+^ Rats but Not in pygm^−/−^ Rats

Consistent with previous findings, we found in pygm^+/+^ rats that lactic acid solution injected in the circulation of the triceps surae muscles with simulated peripheral artery disease evoked a greater pressor response than that when the solution was injected into the contralateral, freely perfused, circulation (Figure 5A,D) [17, 22, 46]. Collectively, our results demonstrate that the magnitude of the metabolic component of the exercise pressor reflex is increased by the simulated peripheral artery disease. As the pH of lactic acid solution approximates 2.6, this augmented response to lactic acid solution can be attributed to both ASIC3 and TRPV1 channels located on the nerve endings of Group III–IV afferents. Both channels are upregulated in models of peripheral artery disease [44, 45, 56], open at that pH [52, 57] and contribute to the pressor response evoked by injection of lactic acid solution [22, 58]. However, it seems unlikely that TRPV1 contributed to the augmented exercise pressor reflex during static contractions in vivo. When muscles contract, intramuscular pH never reaches the threshold required to activate TRPV1 channels (i.e., pH < 5.5) [23, 59, 60, 61] and pharmacological or genetic blockade of TRPV1 function had no effect on the exercise pressor reflex in rats with chronic or acute artery occlusion [10, 42].

Using a more physiologically relevant solution, we replicated the findings of lactic acid experiments by injecting a solution of 24 mM of diprotonated phosphate (pH 6.0). At a pH of 6.0 or above, it is unlikely that TRPV1 played a role in this augmented response. Indeed, a previous report found that pharmacological blockade of TRPV1 had no effect on the pressor response evoked by injection of diprotonated phosphate solution in rats with freely perfused hindlimbs [62]. In addition, Kim et al. [17] found that genetic deletion of ASIC3 reduced the pressor response evoked by the compound in rats with simulated peripheral artery disease. Therefore, we hypothesize that the greater responsiveness of the metabolic component of the exercise pressor reflex induced by chronic femoral artery ligation is arising primarily through ASIC3.

Surprisingly, the pressor response to injection of lactic acid or diprotonated phosphate was not augmented by chronic femoral artery ligation in pygm^−/−^ rats (Figure 5A,D). Previous experiments showed that this increased responsiveness to acidic stimuli was associated with upregulation of ASIC3 receptors [44, 45]. We therefore hypothesize that rendering myophosphorylase non‐functional prevented the sustained muscle acidosis and lactate accumulation induced by chronic femoral artery ligation [63, 64] which blunted the upregulation of acid sensitive channels [44, 45]. For example, Sjöholm et al. [64] showed that a 2.5 h circulatory occlusion progressively decreased muscle pH and increased lactate accumulation which was not restored until the circulatory arrest of removed. Our hypothesis is supported by findings showing that acidosis is a key initiator of the TrkA/nerve growth factor pathway [65, 66], and that nerve growth factor signaling pathways play a key role in (1) upregulating ASIC3 channels [67] and (2) augmenting the metabolic responsiveness of the exercise pressor reflex in peripheral artery disease [68]. Further experiments are needed to test and verify our hypothesis or elucidate the exact mechanisms at play.

### 4.3 Peripheral Artery Disease Exaggerates the Mechanoreflex in Both pygm^+/+^ and pygm^−/−^

Although the metabolic component of the exercise pressor reflex in peripheral artery disease has drawn a lot of attention, less data are available on the mechanoreflex. In line with results from different laboratories [9, 16, 20, 22, 68], we found that in both pygm^+/+^ and pygm^−/−^ rats that the mechanoreflex evoked by passive stretch of the triceps surae muscles whose femoral arteries were chronically ligated was augmented compared to the mechanoreflex evoked from the contralateral, freely perfused hindlimb (Figures 3A,E and 4A,D). Given that the tension between the two maneuvers was not different, these results indicate that the responsiveness of the mechanical component of the reflex was increased by the simulated peripheral artery disease. Multiple studies suggested that mechanoreflex sensitization can result from the accumulation of cyclooxygenase by‐products, such as PGE2, which is produced during muscle stretch [6, 19, 20, 69]. The mechano‐gated channels that could be sensitized by cyclooxygenase by‐products include Piezo 2 [16] and TRPC6 [70], both of which have been identified as key contributors to the mechanoreflex [16, 71, 72, 73]. Importantly, blocking Piezo 2 and TRPC6 with GsMTx‐4 significantly reduced the mechanoreflex evoked by tendon stretch in a rat model of peripheral artery disease [16]. Alternatively, PGE2 can directly activate endoperoxide 4 receptors, a family of channels that produce a pressor response when activated [20]. Our results showing that these receptors are upregulated in dorsal root ganglia from hindlimb muscles with simulated peripheral artery disease support the hypothesis that direct activation of endoperoxide 4 during passive stretch contributes to the exaggerated mechanoreflex in peripheral artery disease.

The fact that the mechanoreflex was exaggerated by peripheral artery disease in both groups contrasts with our results showing that the pressor responses to static contraction or lactic acid injection were not augmented in pygm^−/−^ rats. These findings suggest that the mechanisms responsible for augmenting the mechanical component of the exercise pressor reflex are independent of the mechanisms responsible for augmenting the metabolic component of the reflex. The latter appears to require functional myophosphorylase and most likely sustained acidosis and lactate production whereas the former does not. In support of this hypothesis are findings showing that nerve growth factor contributed to exaggerate the exercise pressor reflex and its metabolic component in a rat model of peripheral artery disease but that nerve growth factor played no role in exaggerating the mechanical component of the reflex [68]. Distinct signaling pathways seem to be involved. If verified, this hypothesis appears to have very important clinical applications as it raises the possibility that treatment could be tailored to selectively target one component or the other to treat the symptoms of peripheral artery disease.

### 4.4 Methodological Considerations

Two key methodological strengths of our protocol design should be considered while interpreting our results. First, the effects of peripheral artery disease were compared to the contralateral leg in each rat, meaning that each rat was its own control. Although previous investigations compared the chronic femoral artery ligation procedure to separate control animals with freely perfused circulation [10, 17], our approach ensured that, among others, variations in surgery, or inter‐animal physiological variability were minimized if not eliminated. In addition, this particular aspect of our experimental design enabled us to precisely quantify the exaggeration of the pressor responses induced by peripheral artery disease and the part of it that was removed by our genetic knock out model. Something that cannot be done with experiments on separate animals. Second, each surgery and experiment were conducted blinded regarding the type of animals (pygm^+/+^ vs. pygm^−/−^). This strengthened our findings by ensuring that conscious or unconscious biases of the experimenter did not influence our results.

We conclude that the lack of an exaggerated exercise pressor reflex in pygm^−/−^ in simulated peripheral artery disease was the result of a lack of hydrogen ions and lactate accumulation. Alternatively, it could have been the result of a lack of upregulation of receptors not related to acidic sensitive channels. For example, two studies showed that endoperoxide 4 receptors are upregulated in peripheral artery disease and that blocking these channels decreased the exercise pressor reflex in rats with simulated peripheral artery disease [19, 20]. However, this hypothesis is not supported by our data showing that endoperoxide 4 remained upregulated in dorsal root ganglia of both pygm^−/−^ and pygm^+/+^ rats. Importantly, these experiments were conducted on a small sample (*n* = 3) and should therefore be interpreted with caution. Further experiments are needed to determine the role played by EP4, and other receptors (e.g., purinergic receptors), in evoking the exercise pressor reflex in rats with non‐functional myophosphorylase with and without simulated peripheral artery disease.

Our conflicting results showing that the pressor response evoked by passive stretch in pygm^−/−^ rats was exaggerated with simulated peripheral artery disease whereas it was not during static contraction question the validity of the passive stretch maneuver as a surrogate for isolating the mechanical component of the exercise pressor reflex evoked by static contraction [25, 71, 73, 74]. Assuming that passive stretch accurately represents the mechanical component of the exercise pressor reflex, it would have been found in pygm^−/−^ rats that the chronic femoral artery ligation still augmented the exercise pressor reflex. One possible explanation could be that the mechanical stimulus of the passive stretch maneuver (i.e., lengthening of the muscle fibers) involves different mechanisms than the mechanical stimulus of statically contracting muscles (i.e., compression muscle fibers). Despite this consideration, it is important to emphasize that passive stretch remains the only maneuver that stimulates mechano‐gated channels without producing metabolic by‐products. In addition, the stretching stimulus might be relevant in the context of movements with eccentric contraction (i.e., lengthening active muscle fibers), a type of contraction that composed numerous daily activities such as walking, running, or decelerating weights.

## 5 Conclusion

Using a unique genetic model that rendered myophosphorylase non‐functional, we provided strong evidence that the lack of lactate and hydrogen ions accumulation prevented an exaggerated exercise pressor reflex and its metaboreflex component in rats with simulated peripheral artery disease. In contrast, we found that the mechanoreflex remained exaggerated regardless of the functionality of myophosphorylase. These results highlight that the mechanisms responsible for exaggerating the metaboreflex are different from the mechanisms responsible for exaggerating the mechanoreflex. Together, our findings raise the possibility that treatments aiming to contain the hemodynamic symptoms of peripheral artery disease could be tailored to target the exaggerated metaboreflex or the exaggerated mechanoreflex.

## Author Contributions

G.P.D. designed the experiments. L.A. acquired the western blot data. G.P.D. acquired, analyzed the data, and drafted the manuscript. All authors interpreted the data and revised the manuscript critically. All authors approved the final version of the manuscript and agree to be accountable for all aspects of the work in ensuring that questions related to the accuracy or integrity of any part of the work are appropriately investigated and resolved. All persons designated as authors qualify for authorship, and all those who qualify for authorship are listed.

## Funding

This study was supported by the National Heart, Lung, and Blood Institute (R01HL161160, R01HL156594, and R01HL156513).

## Disclosure

Artificial intelligence generated content: No content has been generated by an artificial intelligence.

## Conflicts of Interest

The authors declare no conflicts of interest.

## Data Availability

The data that support the findings of this study are available from the corresponding author upon reasonable request.
